# A risk prediction model mediated by genes of APOD/APOC1/SQLE associates with prognosis in cervical cancer

**DOI:** 10.1186/s12905-022-02083-4

**Published:** 2022-12-19

**Authors:** Ya Zhang, Yuankun Qin, Danqing Li, Yingjie Yang

**Affiliations:** 1 Department of Obstetrics and Gynecology, Guizhou Medical University, No.9 Beijing Road, Yunyan District, Guizhou, 550000 Guizhou Province China; 2 Department of Obstetrics and Gynecology, The Affiliated Hospital of Guizhou Medical University, Guizhou, 550025 Guizhou Province, China; 3 Guizhou Medical University, No.9 Beijing Road, Yunyan District, Guiyang, 550001 China; 4 Tthe Affiliated Cancer Hospital of Guizhou Medical University, No.1 Beijing West Road, Guiyang, 550000 Guizhou Province China

**Keywords:** Cervical cancer, Risk prediction model, APOD, APOC1, SQLE, Immune

## Abstract

Cervical cancer is one of the most common gynecological malignancies. Due to the high heterogeneity of cervical cancer accelerating cancer progression, it is necessary to identify new prognostic markers and treatment regimens for cervical cancer to improve patients’ survival rates. We purpose to construct and verify a risk prediction model for cervical cancer patients. Based on the analysis of data from the Gene Expression Omnibus database (GEO) and The Cancer Genome Atlas (TCGA), differences of genes in normal and cancer samples were analyzed and then used analysis of WGCNA along with consistent clustering to construct single-factor + multi-factor risk models. After regression analysis, the target genes were obtained as prognostic genes and prognostic risk models were constructed, and the validity of the risk model was confirmed using the receiver operating characteristic curve (ROC) and Kaplan–Meier curve. Subsequently, the above model was verified on the GSE44001 data validation followed by independent prognostic analysis. Enrichment analysis was conducted by grouping the high and low risks of the model. In addition, differences in immune analysis (immune infiltration, immunotherapy), drug sensitivity, and other levels were counted by the high and low risks groups. In our study, three prognostic genes including APOD, APOC1, and SQLE were obtained, and a risk model was constructed along with validation based on the above-mentioned analysis. According to the model, immune correlation and immunotherapy analyses were carried out, which will provide a theoretical basis and reference value for the exploration and treatment of cervical cancer.

**Supplementary Information:**

The online version contains supplementary material available at 10.1186/s12905-022-02083-4.

## Highlights

- Establishment and Validation of a risk model includes APOD, APOC1 and SQLE;
- APOD, APOC1 and SQLE are related to immunotherapy;
- APOD, APOC1 and SQLE associates with prognosis in cervical cancer

**Supplementary Information:**

The online version contains supplementary material available at 10.1186/s12905-022-02083-4.

## Introduction

Cervical cancer ranks third as one of the most common types of gynecological malignancies to affect females [1–3]. Common treatments for cervical cancer include surgery, radiotherapy, chemotherapy, and immunotherapy [2]. The morbidity and mortality of cervical cancer are significantly declined due to vaccination administration and improved screening in recent years.

However, the prognosis of cervical cancer patients remains poor, as the one-year survival rate for cervical cancer patients is no more than 20%. Cervical cancer is a public health concern worldwide at present [2]. As the importance of risk screening to improve the prognosis of patients with cervical cancer is concerned, the risk factors affecting the prognosis of patients with cervical cancer need to be further explored currently.

Many studies have demonstrated multiple risk prediction models for cervical cancer. Rothberg et al. applied a multivariable model based on an electronic health record of cervical intraepithelial neoplasia grade 2 or higher patients could be more target to screen for cervical cancer [4]. In another study involving 768 cases, a deep-learning neural network model was demonstrated as a useful survival prediction tool in cervical cancer patients [5]. Boruta analysis with the SVM method was confirmed effective for the initial screening of cervical cancer [3]. Human papillomavirus (HPV) is a common risk of cervical cancer according to many studies [6–8]. A variety of unparalleled molecular characteristics of patients with HPV-positive cancer could help prospective medicine of cervical cancer therapy precision precisely [9]. Some genes and their involved pathways such as genes of ABCG2 + PCNA + TDG were found in the microbial community of the cervical to be associated with cervical intraepithelial neoplasia occurrence and progression [10]. Generally speaking, prediction models may play a vital role in the risk prediction of cervical cancer.

Tumor microenvironment including the immune system and hypoxia correlates with tumor progression and therapeutic outcome [11]. It has been verified that the systemic immune-inflammation index was a chemotherapy efficacy predictor for many types of cancers. The pre-treatment immune-Inflammation Index was indicated as an independent predictor of both the prognosis of cervical squamous cell carcinoma prognosis patients and the pathological complete response [12]. It was reported that a prognostic signature consisting of immune-related long non-coding RNAs, such as CTLA-4, PDCD1, and so on, correspondingly predicted risk of cervical cancer and responded to cervical cancer patients’ immunotherapy of mitomycin C and chemotherapeutics of axitinib and docetaxel [13]. Wang et al. found a series of hypoxia or immunity-related genes to establish a prognostic risk model to predict tumorigenesis and development along with chemotherapy sensitivity in cervical cancer [14]. Moreover, DNA methylation plays an important role in tumorigenesis by regulating the tumor microenvironment, a prognostic signature related to DNA methylation and tumor microenvironment was established to predict therapeutic response and clinical outcomes of cervical cancer patients [11]. Metabolism involved lipid, carbohydrate, and energy pathways associated with immune infiltration closely. Therefore, carbohydrate, lipid, and energy pathway-related metabolic reprogramming is indicated as a crucial predictor for invasive cervical carcinoma prognosis due to its correlation with the microenvironment of immune cells [15]. In addition, on account of the immune microenvironment of the tumor and risk model, a ferroptosis-related gene PTGS2 was identified to predict the prognosis of patients with early-stage cervical cancer [16].

Given the importance of genes, especially immune-related genes prediction models for cervical cancer prognosis, we purposed to establish a genes-related prediction model for the prognosis of cervical cancer patients. To analyze the data from the Gene Expression Omnibus database (GEO) and The Cancer Genome Atlas (TCGA), and then construct single-factor accompanied with multi-factor risk models to obtain the immune-related genes associated with prognosis in cervical cancer followed by identification of the prognostic risk model, it was performed based on the GSE44001 data validation. Moreover, immune correlation and immunotherapy analyses were carried out according to the model, which would provide a theoretical basis and targeted program for the investigation and treatment of cervical cancer.

## Materials and methods

### Data sources

Sample data used in the present study were downloaded from the following datasets: TCGA database (https://portal.gdc.cancer.gov) – TCGA-CESC data sets, including 3 cases of normal and 304 cases of cervical cancer samples and survival data were available in 291 of the cancer samples, so the analysis involving survival time was performed in only 291 samples. In addition, the GEO database (https://www.ncbi.nlm.nih.gov/gds) of the two data sets of GSE63514 and GSE44001 was also involved in our study. The GSE63514 dataset including 24 normal samples and 76 cervical cancer samples was used for the analysis of difference and WGCNA. The GSE44001 dataset consisting of 300 cancer samples with available survival data was used to validate the prognostic model.

The inclusion criteria of samples were as follows: (1) Female, aged 18 to 75 (including 18 and 75); (2) Cervical cancer patients with definite diagnosis confirmed by histology and clinical stage IB1-IIA1; (3) With 0 to 1 of the Eastern Collaborative Oncology Group (ECOG) physical status score; (4) The patients should tolerate surgery with organ and hemopoiesis; (5) Contraindications of conventional surgery were excluded. (6) The patients should agree to provide sufficient tumor tissue samples for experimental expression detection. Including archived tumor samples (paraffin blocks or unstained sections whose number meets the testing requirements specified in this study); If no tumor tissue sample is on file, the subject agrees to undergo rebiopsy of the tumor lesion. The exclusion criteria of patients were as follows: (1) Patients received other antitumor therapy (including chemotherapy, molecular targeted therapy, radiotherapy, immunotherapy, monoclonal antibody therapy) within 4 weeks prior to the initiation of study therapy; (2) Patients who cannot tolerate surgery; (3) Patients with meningeal metastasis or symptomatic central nervous system metastasis; (4) Patients with a serious medical condition, such as severe infections, uncontrolled diabetes, cardiovascular disease (New York Heart Association classification of heart failure grade III or IV, heart block grade II or higher, myocardial infarction in the past 6 months, unstable arrhythmias or unstable angina, 3 Cerebral infarction, etc.) or pulmonary disease (history of interstitial pneumonia, obstructive pulmonary disease, and symptomatic bronchospasm) within a month; (5) Patients with other malignant tumors in the previous 5 years before surgery, except for any type of previously cured carcinoma in situ and cured basal cell carcinoma or squamous cell carcinoma of the skin; (6) Patients received allogeneic hematopoietic stem cell transplantation or solid organ transplantation; (7) Patients with a clear history of neurological or mental disorders, such as epilepsy and dementia, and poor compliance; (8) The participants are not suitable for other reasons confirmed by the researchers.

### Genes expression analysis in cervical cancer

To obtain the differentially expressed genes in different groups and conduct further functional mining for the differentially expressed genes, the differential expression score of GSE63514 was analyzed first. Our analysis of data sets GSE63514 differentially expressed difference filter conditions to adjust. *P* < 0.05 and |log2FC| > 0.5. Then, data were analyzed using Weighted Gene Co-expression Network Analysis (WGCNA), which is an analysis method for analyzing gene expression patterns in multiple samples. As modules are distinguished by gene expression similarity, the correlation between modules and modules as well as the correlation between modules and sample traits is calculated, so as to screen highly correlated models and analyze the genes in modules, so as to find the target genes related to the study. In order to check the overall correlation of all samples in the data set, we cluster the samples and eliminate the outlier samples to ensure the accuracy of the analysis. The core of co-expression matrix construction is to classify tens of thousands of genes in the input expression matrix into dozens of modules. In general, we calculated the adjacency between genes and the similarity between genes according to adjacency, then deduce the coefficient of dissimilarity between genes, and get the systematic clustering tree between genes. Then, the minimum number of genes per gene module is set to 30 according to the standard of the dynamic Tree cutting algorithm. To focus on the module and the correlation of disease, therefore, in the screening of the key module, we select the conditions of the module as follows: the grey module, |cor| > 0.3, *P* < 0.05. Moreover, due to our subsequent analysis shall be carried out in the TCGA, so we performed differential expression analysis of differences between the filter to adjust *P* < 0.05 and |log2FC| > 0.5 on the TCGA data set and differential expression of key modules of gene screening along with consensus clustering were carried out accordingly.

### Establishment of a risk prediction model for the prognosis of patients with cervical cancer

To evaluate whether the obtained genes were correlated with the survival of patients survival with cervical cancer, univariate and multifactorial proportional hazards model(COX) analyses was performed in the present study. 291 samples from the TCGA dataset were divided into 7:3 (204:87) as the training set and test set respectively. Univariate Cox proportional risk regression analysis was performed on these genes using the data from the training set to verify whether these genes are risk factors. We set the single-factor cutoff to 0.05. The genes obtained after univariate Cox analysis were then constructed into a multivariate Cox regression model. The multivariate Cox regression algorithm included multiple independent variables (the multiple independent variables here refer to the variables P < 0.05 in the results of univariate Cox regression analysis) into the multivariate Cox analysis, followed by the stepwise regression function step, Parameter direction is set to ‘both’ to adjust the multi-factor regression model.

### Evaluation of the risk prediction model for the prognosis of patients with cervical cancer

To evaluate the prognostic value of the risk model, the risk value of each patient was calculated by analyzing the expression amount of the obtained genes. According to the risk value, the optimal threshold was calculated to divide the patients into two groups of high and low risk. We evaluated risk regression models in a training set (204 samples). To verify the applicability of the model, 300 samples from the GSE44001 dataset were used as the validation set to verify the risk model mainly including the correlation analysis between risk factors and clinical features, the influence of prognostic genes on survival, using a multi-factor model to calculate the risk score, survival receiver operating characteristic curve (ROC) package was applied to calculate false positive and true positive, results of which were used to draw ROC curve and calculate Area Under Curve (AUC) and the whole graph is divided into two parts as the Area below the Curve is called AUC, which is used to indicate the accuracy of prediction. Univariate and multivariate Cox was used to analyze the relationship between clinical traits and patient survival. The nomogram which could predict overall survival (OS) of cervical cancer patients with prognostic genes and other clinical traits with *p* < 0.05 was established and assessed using a calibration curve and decision curve analysis (DCA) curve.

### Pathway analysis of the risk prediction model for prognosis of patients with cervical cancer

The risk values were divided into high and low-risk groups at the optimal threshold, and the Limma package was used to conduct difference analysis on samples from high and low-risk groups. To further explore the difference between high and low-risk groups, we sorted the log2FC values from high to low and then conducted Gene Set Enrichment Analysis (GSEA) enrichment analysis. In this project, the R software cluster Profiler package was used for GSEA enrichment analysis to search for common functions and related pathways of a large number of genes in the differentially expressed gene set. The enrichment analysis databases used by GSEA include the Kyoto Encyclopedia of Genes and Genomes (KEGG) and Gene Onotology (GO).

### Immune correlation analysis based on the risk prediction model for the prognosis of patients with cervical cancer

Single-sample GSEA (ssGSEA) algorithm was used to infer immune cell abundance. Through ssGSEA, we can get the immune cells or immune function of each sample and the activity of immune pathways, and then group them according to the immune activity. We used the Immunoapparent score (IPS) method to calculate IPS from all patient samples. IPS is an indicator to measure the overall immunogenicity of the tumor, which mainly includes the following four categories: Effector cells, immunosuppressive cells, MHC molecules and immunomodulators. IPS can unbiasedly consider using machine learning to determine immunogenicity based on four categories of genes.

### Analysis of immunotherapeutic response and prediction of chemotherapeutic drug sensitivity based on the risk prediction model for prognosis of patients with cervical cancer

It is an important purpose of immunotherapy to reactivate immune cells and reverse the immunosuppressive state of the tumor microenvironment. We use the Tumor Immune Dysfunction and Exclusion (TIDE, http://tide.dfci.harvard.edu/) to predict response to immunotherapy.

The Cancer Drug Sensitivity Genomics (GDSC) database is the largest public resource for information on molecular markers of drug sensitivity and drug response in cancer cells. It contains extensive drug sensitivity and genomic data sets that are important for the discovery of potential tumor therapeutic targets. PRRopheticPredict package (version 0.5) of R language was used to calculate 138 drugs included in the database, and significant differences of IC50 among different groups were obtained through calculation in our study.

### Real-time quantitative PCR (qPCR) detection of genes

Ten samples of cervical cancer tissue and ten samples of corresponding tissue adjacent to cervical carcinoma were obtained from patients at the Affiliated Cancer Hospital of Guizhou Medical University, and each patient wrote informed consent. The total RNA of the samples was isolated using TRIzol Reagent (REF:15,596,018) from ambion. All-in-onetM first-strand cDNA Synthesis Kit (REF:15,596,018) was used for reverse transcription reaction and PCR was performed using the 2xUniversal Blue SYBR Green qPCR Master Mix kit (G3326-05) provided by Servicebio. Reaction program was performed with 40 cycles including pre-denaturation at 95 °C for 1 min, denaturation at 95 °C for 20 s, annealing at 55 °C for 20 s, and extension at 72 °C for 30 s. The primers were shown in Table 1. RNA (Fold Change vs. Cont) = 2^−∆∆CT. Results of qPCR were analyzed using GraphPad Prism 9 (USA). The paired T-test was used for difference analysis, when the p value was less to 0.05 the difference was significant.

**Table 1:** Primers for real-time quantitative PCR

Gene	Primers (5′–3′)
APOC1 F	CCTGGTGGTGGTTCTGT
APOC1 R	CTCTGTTTGATGCGGCT
SQLE F	TACTGGGCAAGAAAAACAT
SQLE R	ACCACTACTGAGAAGGGCT
APOD F	GGCAGAGGGACAAGCATT
APOD R	CTGGAGGGAGATTAGGGT
GAPDH F	CCCATCACCATCTTCCAGG
GAPDH R	CATCACGCCACAGTTTCCC

## Results

### Differentially expressed genes obtained from screening

**Fig. 1:**
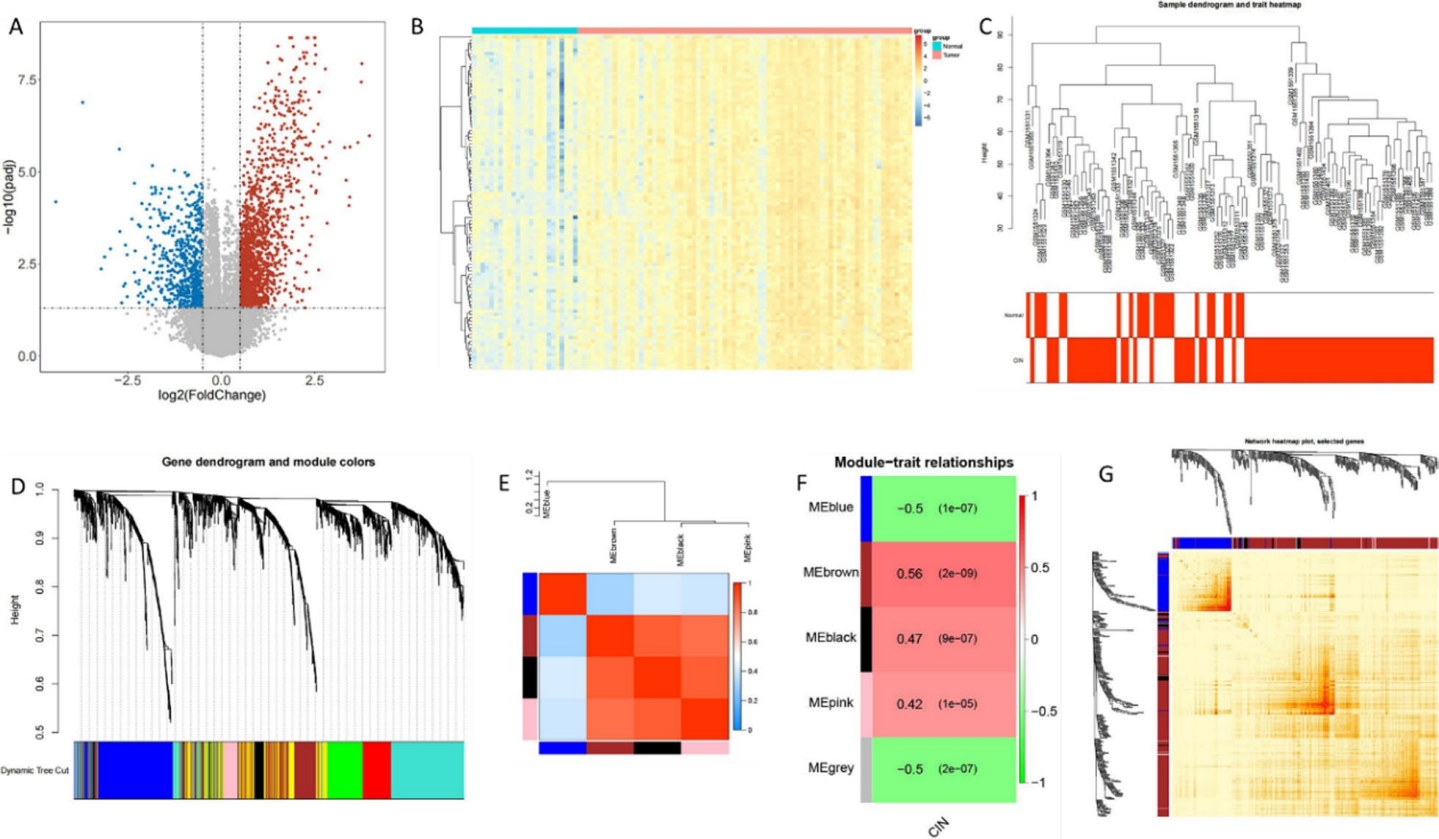
Differential expression of genes analyzed on GSE63514 data set. **a** Volcano map of genes of caner tissue compared with normal samples. **b** Heat map top 100 differentially expressed genes between caner tissue and normal samples. **c** The sample clustering and heat maps of clinical traits to exhibit the overall correlation of all samples in the dataset. **d** The module diagram of co-expression matrix with systematic clustering tree between genes. **e** Module and character association diagram with clustering tree and heat map. f Heat map of correlation between different modules and clinical traits. **g** Cluster diagram of topological matrix containing 40 genes

Differential expression analysis was performed on the GSE63514 data set. According to statistics, a total of 2339 genes were significantly differentially expressed between normal samples and cancer patients, including 1742 up-regulated genes and 597 down-regulated genes. A volcano map of genes of cancer tissue compared with normal samples was showed in Fig. 1a. Top 100 differentially expressed genes between caner tissue and normal samples were showed in the heat map (Fig. 1b). In order to further identify genes closely related to diseases, WGCNA analysis was performed on 2339 differential genes in the GEO dataset (GSE63514), and diseases in the GEO dataset were used as traits for WGCNA analysis. For the overall correlation of all samples in the dataset, we first cluster the samples and the sample clustering and heat maps of clinical traits was showed in Fig. 1c. We constructed the co-expression matrix to obtain the systematic clustering tree between genes. MEDissThres is set to 0.2 to merge the similar modules analyzed by the dynamic clip-tree algorithm. After merging, there are finally 5 modules (one of them is gray, indicating that genes are not classified into any module), and the following module diagram is generated (Fig. 1d). As modules correlated with clinical features as concerned, module and character association diagram and heat map were showed in Fig. 1e, f. In this study, we focused on the correlation between modules and diseases, 400 genes were randomly selected for the gene cluster tree and heat map (Fig. 1g).

**Fig. 2:**
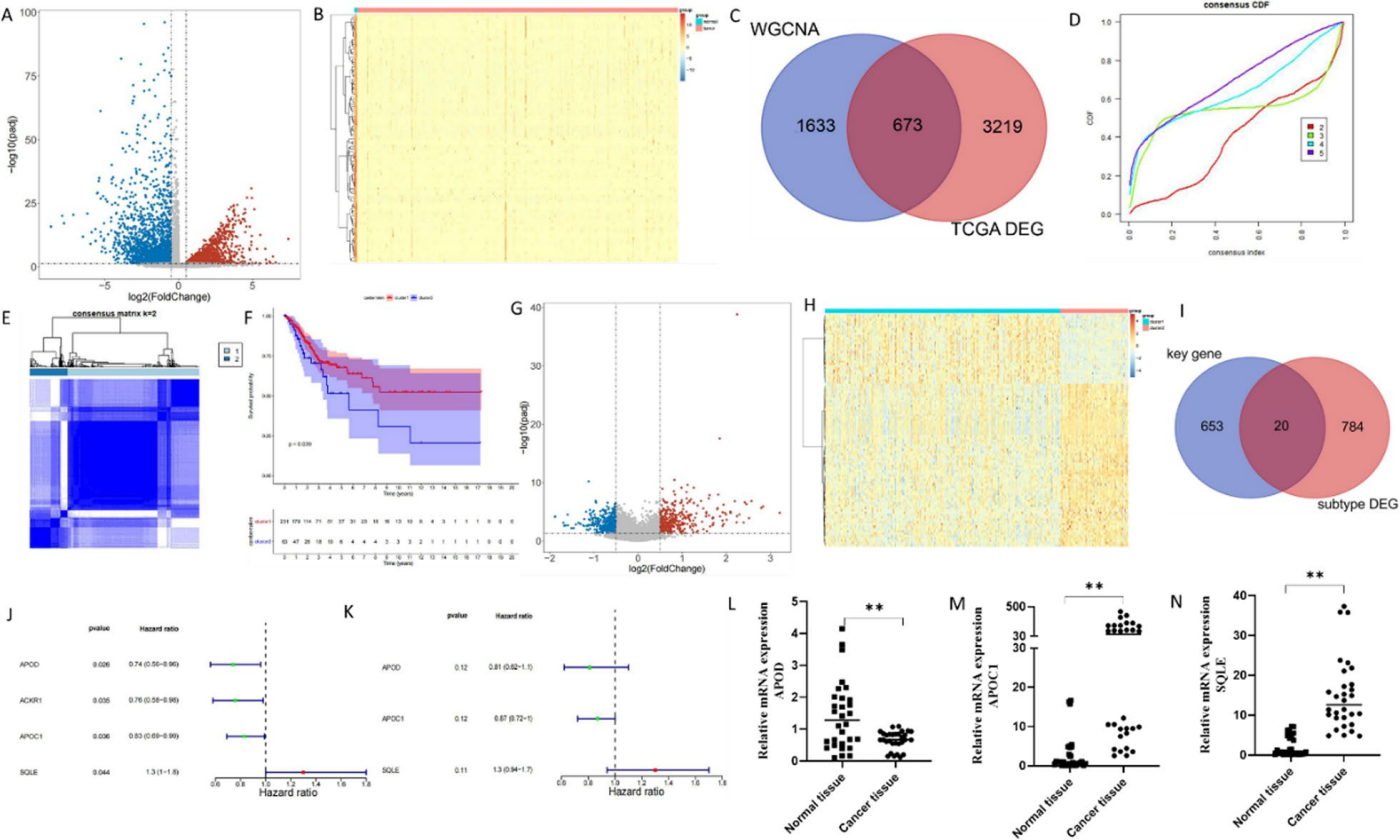
Differential expression of genes analyzed on TCGA data set. **a** Volcano map of genes between caner tissue compared and normal samples. **b** Heat map top 100 differentially expressed genes in caner tissue compared with normal samples. **c** Venn diagram of key gene modules differentially expressed. **d** Consistent clustering accumulative distribution function (CDF) diagram. **e** Heat map of consistent clustering. **f** Kaplan–Meier curves of cancer patients with different clustering subtypes. **g** TCGA samples were divided into Cluster1:236 cases and Cluster2:68 cases. Volcano map of Cluster2 compared with Cluster1 sample gene. h Heat map of Cluster2 compared with Cluster1 samples. **i** Venn diagram of differential genes and key genes between subtypes. **j** Forest map of univariate COX results. **k** Forest map with multivariate COX results. **l** The mRNA expression of APOD assayed by qPCR in 10 samples of cervical cancer tissue and normal tissue. **m** The mRNA expression of APOC1 assayed by qPCR in 10 samples of cervical cancer tissue and normal tissue. **n** The mRNA expression of SQLE assayed by qPCR in 10 samples of cervical cancer tissue and normal tissue. n = 10. **P = 0.01 compared with normal tissue

Differential expression analysis of the TCGA data set indicated that 3892 genes were significantly differentially expressed between normal samples and cancer patients, including 1660 up-regulated genes and 2232 down-regulated genes, and the Volcano Plot along with the heat map of the expression of the top100 differentially expressed genes were showed in Fig. 2a, b. To screen out differentially expressed key module genes in TCGA and GEO, we obtained 3892 differential genes and 2306 key module genes in TCGA and obtained 673 intersection genes by the intersection of the two gene sets, which were defined as key genes. For downstream analysis, a VENN diagram is shown in Fig. 2c. Consistent cluster analysis was performed using the expression data of 673 key genes in 304 cancer samples from TCGA. Consistent clustering accumulative distribution function (CDF) diagram and consistent clustering heat map are shown in Fig. 2d, e. Next, we conducted survival analysis based on different cluster subtypes and survival information of cancer patients, and the results are shown in the Fig. 2f. It can be seen that the overall survival time of the two categories is significantly different, and the survival probability of Cluster1 patients is higher. The TCGA samples were divided into Cluster1: the 236 cases, Cluster2:68 examples, we have analyzed differentially expressed between different subtypes. According to statistics, 804 genes were significantly differentially expressed between Cluster1 samples and Cluster2 patients, including 395 up-regulated genes and 409 down-regulated genes (Fig. 2g, h). To screen out different genes between subtypes, we obtained 673 key genes and 804 differential genes between subtypes, and the intersection of the two gene sets was used to obtain 20 overlapping genes for downstream analysis, as shown in the Venn diagram (Fig. 2i). In order to evaluate whether the 20 genes are associated with patient survival, 291 samples from the TCGA dataset were divided into 7:3 (204:87) as training set and test set respectively. Univariate Cox proportional risk regression analysis was performed on these genes using the data from the training set to verify whether these genes are risk factors and four genes were obtained (Table 2; Fig. 2j). After multivariate Cox analysis, a total of 3 genes appeared in the multivariate Cox analysis results, including APOD, APOC1, and SQLE, which were taken as prognostic factors of this study (Table 3; Fig. 2k). The expression levels of the prognostic factors were plotted in Additional files 1 and 2: Figures S1 and S2. Simultaneously, the mRNA expression of APOD, APOC1, and SQLE was further assayed in cervical cancer tissues and normal tissues. The results, as shown in Fig. 2l–n, illustrated that APOD was significantly down-regulated while the gene expressions of APOC1 and SQLE were higher in cancer samples compared with normal samples, which was consistent with that in online databases.

**Table 2:** Results of univariate analysis

ID	z	HR	HR.95 L	HR.95 H	*P* value
APOD	−2.23279	0.737162	0.564038	0.963424	0.025563
ACKR1	−2.1105	0.755724	0.582643	0.980221	0.034815
APOC1	−2.10039	0.827697	0.6938	0.987437	0.035695
SQLE	2.015182	1.341524	1.008083	1.785256	0.043886

**Table 3:** Results of multivariate analysis

ID	z	HR	HR.95 L	HR.95 H	*P* value
APOD	−0.21299	0.808161	0.617038	1.058482	0.121833
APOC1	−0.14381	0.866049	0.723332	1.036926	0.117513
SQLE	0.246593	1.279659	0.943892	1.734866	0.112266

### Establishment and validation of risk prediction model for prognosis of patients with cervical cancer

**Fig. 3:**
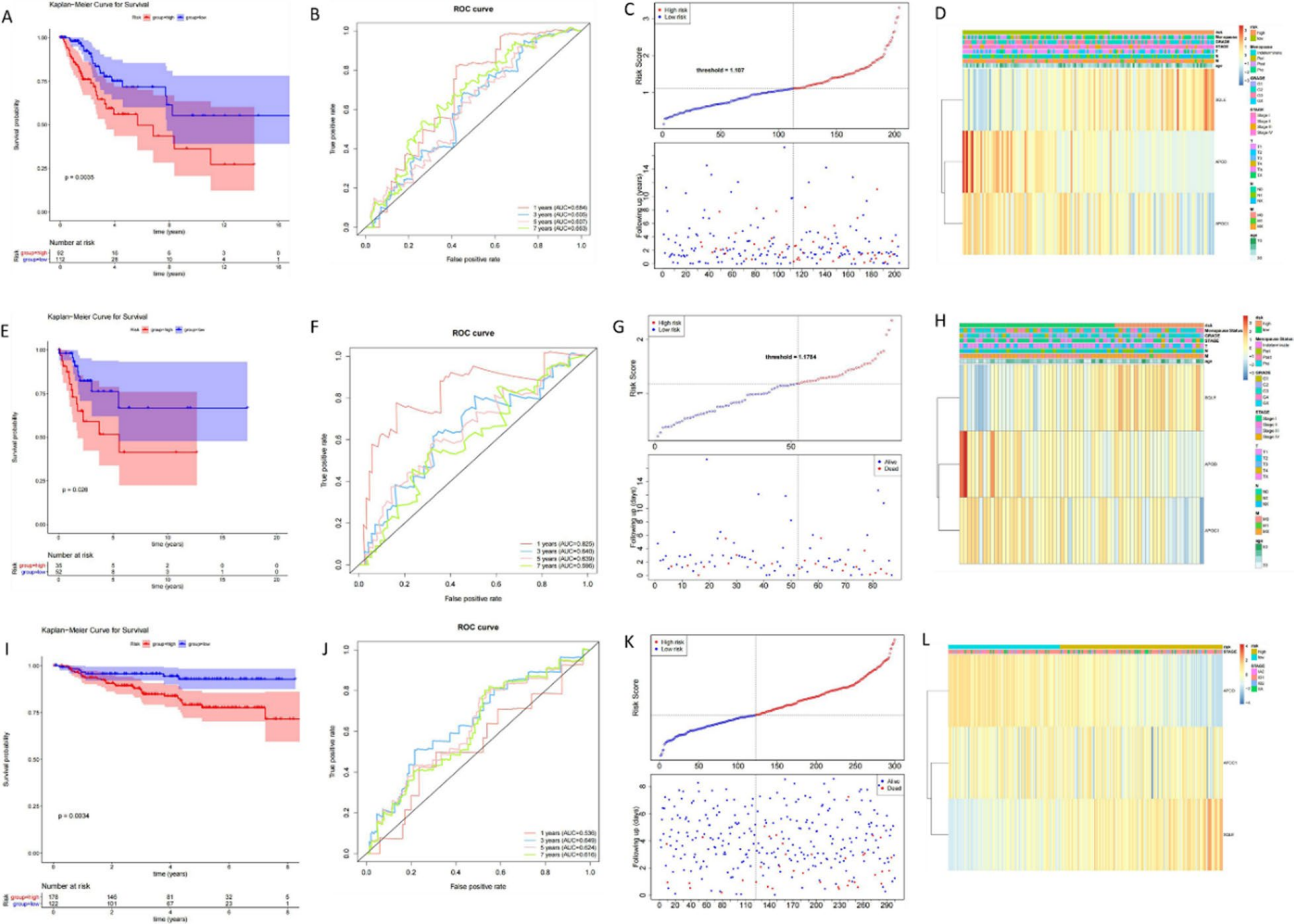
Establishment and validation of prediction model including three genes of APOD, APOC1 and SQLE risk for prognosis of patients with cervical cancer. Risk regression model evaluation was performed. **a** Kaplan–Meier survival curve of Risk score. **b** To evaluate the validity of the risk model using the ROC curve. **c** Risk curves for the high and low risk groups. **d** Heat map of overview of correlation between Risk Score and clinical features. Risk model testing as follows. **e** Kaplan–Meier survival curve of test set-risk score. **f** Test suite-ROC curve to assess the effectiveness of the risk model. **g** Test set - Risk curves for high and low risk groups. **h** Test set profile of the correlation between Risk Score and clinical features. Validation of risk model was performed. **i** Kaplan–Meier survival curve of verifying set-risk score. **j** Validate set-ROC curve to evaluate the validity of risk model. **k** Validation set - Risk curves for high and low risk groups. **l** Overview of the correlation between risk score and clinical features

Based on three genes of APOD, APOC1, and SQLE, the risk prediction model for the prognosis of patients with cervical cancer was established, tested and validated. We evaluated risk regression models in a training set (204 samples). The risk value of each patient was calculated by the expression levels of three genes, and the optimal threshold was calculated according to the risk value to divide the patients into two groups of high and low risk. From the survival analysis of the high-low-risk group (Fig. 3a), it can be seen that there is a significant difference in the survival of the high-low-risk group (*P* < 0.05). ROC Curve was drawn using the results and AUC was calculated (Fig. 3b). The whole graph of AUC was divided into two parts. The higher the AUC value is, that is, the larger the area under the curve (and the smoother the curve), the higher the prediction accuracy will be. The closer the curve is to the top left corner (the smaller the X, the larger the Y), the higher the prediction accuracy. The AUC of 1, 3, 5 and 7 years were all greater than 0.6, indicating that the constructed risk regression model could effectively serve as a prognostic model. The risk curve is composed of the upper and lower graphs (Fig. 3c). The abscissa of the two graphs is consistent, and the risk value of patients increases successively from left to right. The samples were divided into high and low risk groups according to the optimal threshold. We proposed the available clinical data in the data set and displayed them in the heat map(Fig. 3d). Risk was combined with clinical data of patients in Table 4, and the chi-square test was used to evaluate the correlation between the risk model and clinical traits. *P* < 0.05 means that risk has significant differences among different groups. As can be seen from the table, M, N, T and GRADE traits were significantly different between high and low-risk groups. Analogously, the risk prediction model for the prognosis of cervical cancer was tested (Fig. 3e–h) and validated (Fig. 3i–l).

**Table 4:** Risk is correlated with clinical traits of patients

	Total (N = 142)	Risk		
		High (N = 59)	Low (N = 83)	*P* value
Age (years)				
≥60	29 (20.4%)	14 (23.7%)	18 (18.1%)	0.54
<60	113 (79.6%)	45 (76.3%)	68 (81.9%)	
M				
M0	66 (46.5%)	17 (28.8%)	49 (59.0%)	0.00162
M1	8 (5.6%)	5 (8.5%)	3 (3.6%)	
MX	68 (47.9%)	37 (62.7%)	31 (37.3%)	
N				
N0	71 (50.0%)	25 (42.4%)	46 (55.4%)	0.00154
N1	35 (24.6%)	10 (16.9%)	25 (30.1%)	
NX	36 (25.4%)	24 (40.7%)	12 (14.5%)	
T				
T1	77 (54.2%)	25 (42.4%)	52 (62.7%)	0.0129
T2	38 (26.8%)	16 (27.1%)	22 (26.5%)	
T3	11 (7.7%)	5 (8.5%)	6 (7.2%)	
T4	5 (3.5%)	5 (8.5%)	0 (0.0%)	
Tis	8 (5.6%)	5 (8.5%)	0 (0.0%)	
TX	10 (7.0%)	7 (11.9%)	3 (3.6%)	
Stage				
Stage I	83 (58.5%)	32 (54.2%)	51 (61.4%)	0.169
Stage II	32 (22.5%)	15 (25.4%)	17 (20.5%)	
Stage III	17 (12.0%)	5 (8.5%)	12 (14.5%)	
Stage IV	10 (7.0%)	7 (11.9%)	3 (3.6%)	
Grade				
G1	9 (6.3%)	3 (5.1%)	6 (7.2%)	0.00229
G2	63 (44.4%)	26 (44.1%)	37 (44.6%)	
G3	58 (40.8%)	19 (32.2%)	39 (47.0%)	
GX	12 (8.5%)	11 (18.6%)	1 (1.2%)	
Menopause				
Indeterminate	1 (0.7%)	1 (1.7%)	0 (0.0%)	0.117
Peri	16 (11.3%)	3 (5.1%)	13 (15.7%)	
Post	47 (33.1%)	23 (39.0%)	24 (28.9%)	
Pre	78 (54.9%)	32 (54.2%)	46 (55.4%)	

Moreover, results of univariate and multivariate COX analysis showed that risk score and T were significant independent prognostic factors (Tables 5 and 6, Additional file 3: Figure S3). Hence the nomogram and corresponding calibration curve were pictured in Additional file 4 and 5: Figures S4 and S5, indicating that it had a good predicted capability for 3-year OS of patients with cervical cancer. DCA analysis confirmed the clinical validity of the nomogram (Additional file 6: Figure S6). Likewise, risk score also had significant independent prognostic power in the external validation set.

**Table 5:** Results of independent prognostic—univariate analysis

Variable	Coef.	HR	HR.95 L	HR.95 H	*P* value
T	0.60209	1.825931	1.366611	2.439629	4.65E−05
STAGE	0.391985	1.479915	1.189982	1.840489	0.000426
Riskscore	0.657107	1.929203	1.299253	2.864589	0.001122
N	0.9396	2.558958	1.30283	5.026186	0.006372
M	1.267485	3.551909	1.195447	10.55343	0.022532
age	0.015432	1.015552	0.998192	1.033214	0.079386
Menopause	0.082692	1.086208	0.805541	1.464665	0.587699
GRADE	−0.04339	0.957536	0.628443	1.458961	0.839951

**Table 6:** Results of independent prognostic—multiple analysis

Variable	coef	HR	HR.95 L	HR.95 H	*P* value
Riskscore	1.364655	3.914371	1.219048	12.56907	0.021863
T	0.76528	2.149597	1.011026	4.570375	0.046761
STAGE	−0.71097	0.491167	0.224645	1.073892	0.074856
N	0.880091	2.41112	0.869176	6.688521	0.090908

### Pathways involved in the risk prediction model for prognosis of patients with cervical cancer

**Fig. 4:**
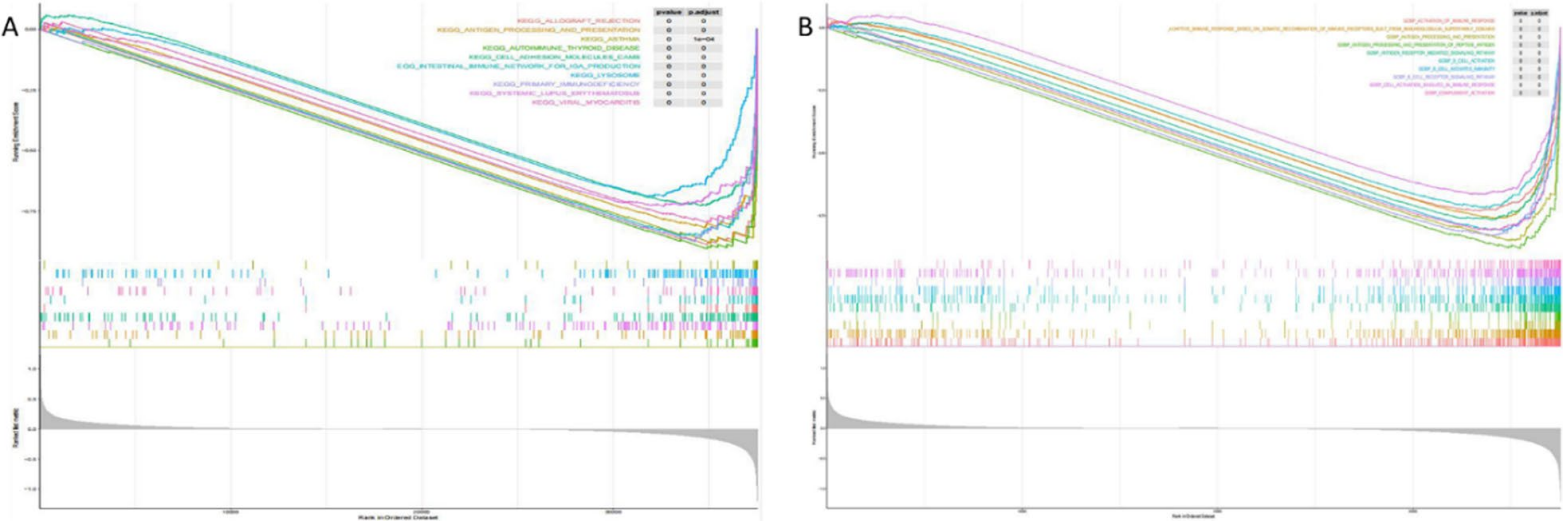
Pathway involved in the risk prediction model for prognosis of patients with cervical cancer. **a** The top10 KEGG pathways were enriched by high and low risk groups. **b**Top10 GO pathways enriched by high and low risk groups

The GO function of differentially expressed genes was annotated and the biological significance of each gene was explored. A total of 21 KEGG pathways were enriched in GSEA [17–19]. The KEGG pathways were visualized as follows, the ordinate in Fig. 4a represents the enrichment score. ES is positive, indicating that a certain functional gene set is enriched in the front of the sequence. ES is negative, indicating that a certain functional gene set is enriched in the rear of the sequence. The horizontal axis represents the gene, and each little vertical line represents a gene. On the whole, it can be seen that this pathway or GO has an upward/downward trend. A total of 748 GO pathways were enriched, and the top10 GO pathways were selected for visualization (Fig. 4b).

### Immune correlation of the risk prediction model for prognosis of patients with cervical cancer

**Fig. 5:**
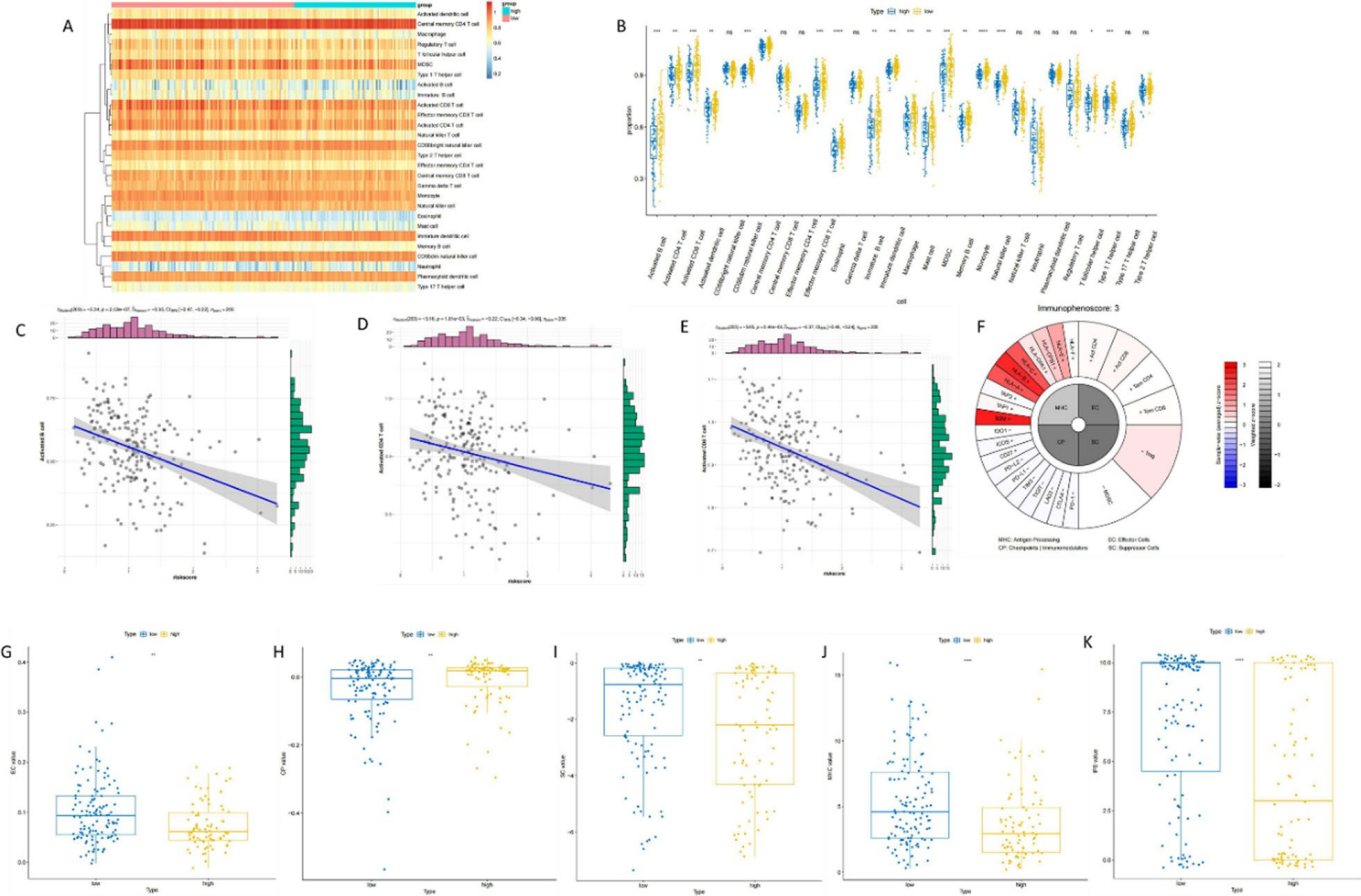
Immunocorrelation of the risk prediction model for prognosis of patients with cervical cancer. **a** Heat maps of different cell content inferred by ssGSEA. **b** Boxplots of ssGSEA inferred cell content between high and low risk groups. **c** Scatter plot of the correlation between Activated B cell and Risk Score. **d** Scatter plot of the correlation between Activated CD4 T cell and Risk Score. **e** Scatter plot of the association between Activated CD8 T cell and Risk Score. **f** IPS pie chart of TCGA-2 W-A8YY-01 A-11R-A37O-07 sample. **g** EC value between high and low risk groups. **h** CP value boxplot between high and low risk groups. **i** The boxplot of SC value between high and low risk groups. **j** Boxplot of MHC value between high and low risk groups. **k** The boxplot of IPS values between high and low risk groups

We obtained the immune cells with immune functions of each sample and the activity of immune pathways through ssGSEA analysis, and then group them according to the immune activity. 28 immune-related gene sets were used, which not only included immune cell types, but also immune-related pathways and immune-related functions. The heat map of different cell content calculated by ssGSEA was shown in Fig. 5a. The box plot of cell content calculated by ssGSEA between high and low-risk groups was shown in Fig. 5b. Among all the 28 cell species, 18 species showed significant differences between groups (rank-sum test). Next, we explored the correlation between immune cells and RiskScore, conducted correlation analysis, and visualized it as a scatter plot. Only three graphs were shown in Fig. 5c–e, and the other 25 types of immune cells were shown in Additional file 7: Figure S7.

IPS was calculated on a scale of 0–10 based on the representative cell type gene expression Z-score, and the higher the score, the stronger the immunogenicity. The IPS method was used to calculate IPS for all patient samples. One of the pie charts of the samples is shown in Fig. 5f. We calculated the differences between the four parts (EC, SC, MHC and CP) and the population (IPS) of the high-low risk group, and visualized them as a box plot (Fig. 5g–k). Results demonstrated significant differences in EC, SC, MHC, CP, and overall (IPS) values between high and low-risk groups.

### Immunotherapeutic response and chemotherapeutic drug sensitivity correlate with the risk prediction model for the prognosis of patients with cervical cancer

**Fig. 6:**
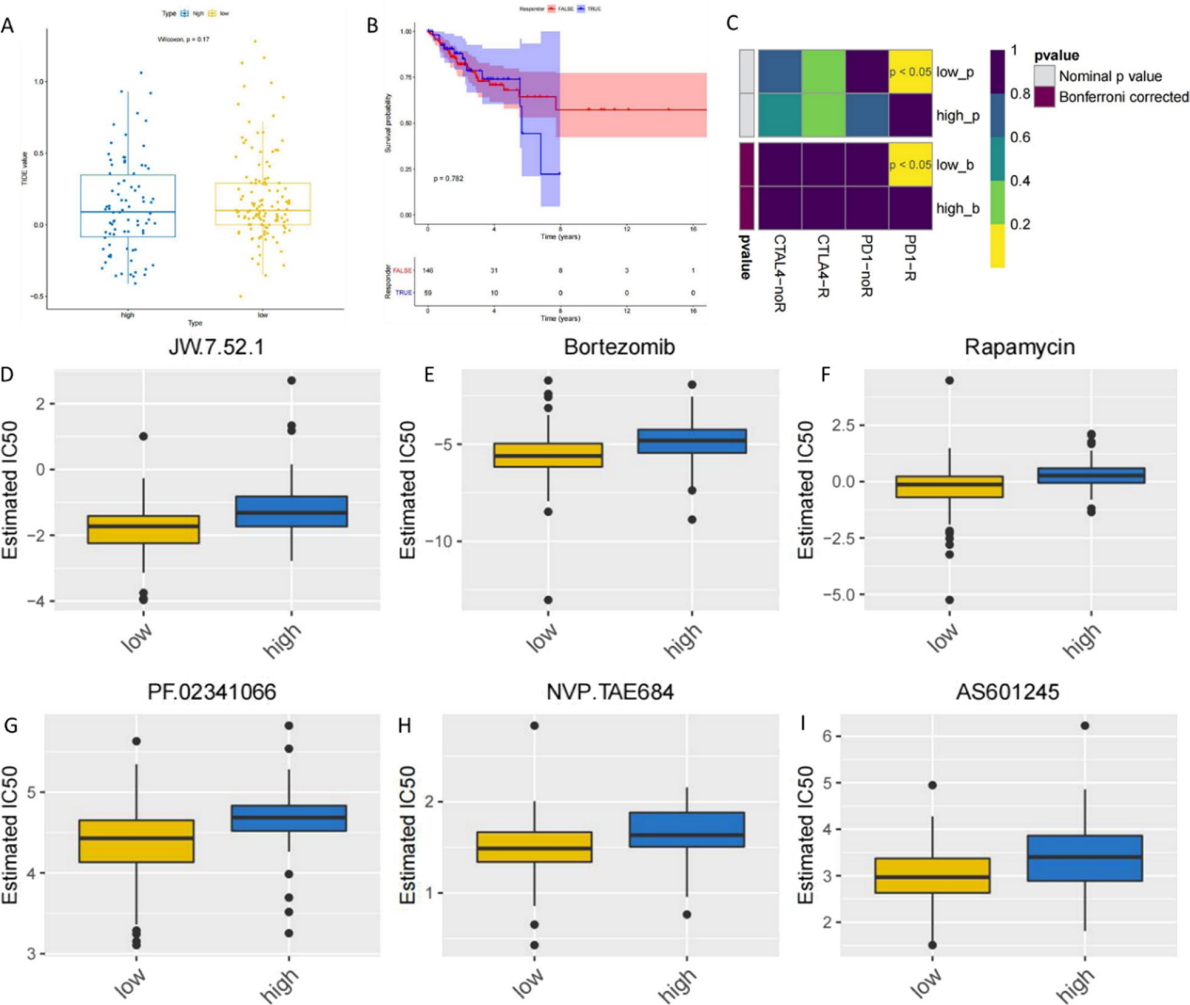
Immunotherapeutic response and chemotherapeutic drug sensitivity correlates with the risk prediction model for prognosis of patients with cervical cancer. **a** Boxplot of TIDE predicting response to high-low subgroups of immunotherapy. **b** Survival curves of cancer patients with different clustering subtypes. **c** SubMap algorithm predicts the likelihood of response to immunotherapy in the high and low risk groups. **d** The difference of sensitivity to JW.7.52.1 between the high and low risk groups was significant. **e** The difference of sensitivity to Bortezomib between the high and low risk groups was significant. **f** The difference of sensitivity to Rapamycin between the high and low risk groups was significant. **g** The difference of sensitivity to PF.02341066 between the high and low risk groups was significant. **h** The difference of sensitivity to NVP.TAE684 between the high and low risk groups was significant. **i** The difference of sensitivity to AS601245 between the high and low risk groups was significant

TIDE was used to predict the likelihood of response to immunotherapy. After TIDE analysis, the predicted likelihood of response to immunotherapy was not significant (Fig. 6a). Survival analysis was conducted based on the response to immunotherapy and survival information of cancer patients, and the results are shown in Fig. 6b, it can be seen in which that there is no significant difference in the overall survival time of samples with different immunotherapy responses. We then used the SubMap algorithm to predict the likelihood of a single sample or subtype responding to immunotherapy. The basic principle is to resist the immunosuppressive effect of the tumor microenvironment by targeting the immune checkpoint receptors—CTLA4, PD1 and their ligands (PDL1, PDL2), so as to remove the immune suppression and enhance the immune function to play an anti-tumor role. As shown in Fig. 6c, low and high are high-low risk groups, low risk is more likely to be more sensitive to anti-PD1 therapy (Nominal *P* and corrected *P* were both less than 0.05).

Through calculating and analyzing 138 chemotherapy drugs contained in the database, the significant difference in median inhibitory concentration (IC50) between different groups could be obtained through calculation. According to the calculation results, a total of 41 drugs showed significant differences in the high and low risk groups, which were visualized as a boxplot as shown in Fig. 6d–i, in which only 6 drugs were shown, and all the 41 significant drugs were shown in Additional file 8: Figure S8.

## Discussion

Cervical cancer is a malignant tumor in women. Conventional treatment of cervical cancer includes radiotherapy, chemotherapy and surgery currently, but progress of tumors in patients are prone to chemoradiotherapy resistance [20]. In addition, the high heterogeneity of cervical cancer accelerates the process of cancer development [21]. Many researchers devote oneself to find new prognostic markers and treatment regimens for cervical cancer. Peritoneal HPV-DNA test in cervical cancer could server a potential clinical implication of prognosis [22]. Some markers such as E-cadherin, Ki67, CEA, and CD44 were reported to server detecting invasive forms of cervical cancer, which might be useful in evaluation and monitoring of treatments of patients with evaluation and monitoring of treatments [23]. Moreover, it remains necessary to identify new prognostic markers and treatment regimens for cervical cancer to improve the survival rate of cervical cancer patients at present.

Immunotherapy has shown great potential in treating cancer in recent years. Immunotherapy consisting of anti-CTLA4 and anti-PD1 agents has been reported to be effective in the treatment of oropharyngeal cancer and cervical cancer. Immune checkpoint inhibitors (ICIs) can treat advanced chemotherapy-resistant cervical cancer. Immune cells and cytokines secreted in the immune microenvironment can inhibit the development of cervical cancer [24]. In summary, response to the immune system in patients with cervical cancer affects tumor progression and treatment. However, there are few therapeutic immunodetection sites, and the research on tumor immunotherapy is far from enough. Therefore, we mainly screened immune-related genes (IRGs) in cervical cancer, constructed a risk model based on these genes, and explored the possible molecular mechanism of cervical cancer in the present study.

In our study, we firstly downloaded GSE63514 data from the GEO database, and the differences between normal and cancer samples were analyzed, then 2339 differential genes were obtained, which were analyzed using WGCNA and 4 modules were screened, including 2306 key module genes. WGCNA is an analysis method for analyzing gene expression patterns in multiple samples, which can cluster the genes with similar expression patterns [25]. The analysis module is widely used in the study of the association between disease and other traits and genes because of its association with specific traits or phenotypes. Many articles use this method to find potential biomarkers and drug targets [25–27]. Then, modules are distinguished through gene expression similarity, and then the correlation between modules and modules as well as the correlation between modules and sample traits is calculated, so as to screen highly correlated models of traits, and analyze the genes in modules, so as to find the target genes related to our study. Afterwards we download the TCGA data for differences analysis in normal and cancer samples to acquire 3892 different genes, then gene takes overlap with the key module and got 673 intersection genes. 673 overlapping genes were used for consistent clustering in TCGA cancer samples, which were divided into two categories: Cluster1 contains 236 cases, Cluster2 contains 68 cases, and two subtypes have significant differences in survival. A total of 804 differential genes were obtained. The intersection of 673 intersected genes and 804 differentially differentiated genes between subtypes was selected for 20 intersected genes for further being used to construct single-factor + multi-factor risk models. After regression analysis, 3 genes such as APOD, APOC1 and SQLE were obtained as prognostic genes and prognostic risk models were constructed. According to previous studies, APOD was an immune-related gene and is associated with the risk of multiple types of cancer such as gastric cancer [28], thyroid cancer [29], breast cancer and cervical cancer [30], which was also considered as one of prognostic signatures in cancer. APOC1 was reported as an oncogene to promote tumorigenesis and progression of glioblastoma [31], gastric cancer [32], hepatocellular carcinoma [33], breast cancer [34] and so on. SQLE is a key enzyme of cholesterol biosynthesis [35] and tends to be over-expressed along with treatment sensitivity in colon cancer [36], breast cancer and non-small cell lung cancer [37], Acute Myeloid Leukemia [38] and pancreatic cancer [39]. SQLE associates prognosis prediction of patients with bladder cancer [40]. Based on the differential genes screened, the expression of APOD, APOC1 and SQLE was also measured in 10 samples of cervical cancer tissue and para-cancer normal tissue in our study, results of which demonstrated that APOD expression was lower as well as APOC1 and SQLE were higher in cancer samples than normal samples.

To explore the risk prediction signature in cervical cancer, a three-genes of APOD, APOC1 and SQLE-involved risk model was constructed, tested and verified in our further study. By drawing the ROC curve and Kaplan–Meier curve, the validity of the risk model (AUC > 0.6) was confirmed. Subsequently, this model was verified on the GSE44001 data validation set and verified (AUC > 0.6), indicating that the risk model constructed by us can effectively predict the prognosis of the disease. In addition, in order to verify whether the risk model can accurately predict patient survival rate, we conducted an independent prognostic analysis of the risk model and found that the risk model can accurately predict patient survival rate.

In order to investigate why the model can effectively predict the prognosis of patients, we conducted an enrichment analysis of GSEA based on the high and low-risk groups of the model. The enrichment analysis databases GSEA includes KEGG and GO. KEGG is the Kyoto Encyclopedia of Genes and genomes, designed to understand advanced functional and biological systems (such as cells, organisms and ecosystems), from molecule-level information, especially from large molecular data sets generated by genome sequencing and other high-throughput experimental techniques of the utility database resources, which is one of the most commonly used bioinformatic databases in the world and is known as “a repository of advanced functions and utilities for understanding biological systems“ [41]. The GO system consists of three parts: Biological process, molecular functions, and cellular components [42]. We annotated the GO function of differentially expressed genes and explored the biological significance of each gene.

Moreover, we analyzed the differences in immunoassay (immune infiltration, immunotherapy) and drug sensitivity at different levels in the high and low-risk groups. Through SsGSEA method, we can get the immune cells or immune function of each sample and the activity of immune pathways, and then group them according to the immune activity SsGSEA is an implementation method proposed mainly for a single sample that cannot do GSEA. IPS is an indicator to measure the overall immunogenicity of tumors, which is used in our study and can determine immunogenicity without bias using machine learning based on four categories of genes. Immunotherapy is to artificially enhance or inhibit the immune function of patients with cancers. Tumor immunotherapy aims to activate the body’s immune system to kill pathogenic factors (bacteria, fungi, cancer cells or tumor tissue) by its own immune function. Unlike previous surgery, chemotherapy, radiotherapy and targeted therapy, immunotherapy targets the body’s own immune system rather than tumor cells and tissues. Therefore, it is an important goal of immunotherapy to reactivate immune cells and reverse the immunosuppressive state of the tumor microenvironment. Therefore, we used the TIDE [43] to predict the response to immunotherapy and SubMap, an unsupervised approach to estimate the associations between subtypes observed in two independent data sets, to predict the response to immunotherapy for a single sample or subtype in the present study.

Drug sensitivity correlates with patient prognosis closely, therefore, the GDSC database was used to evaluate the differences in drug sensitivity between the high and low-risk groups divided by the risk prediction mode in our study. The GDSC database is the largest public resource for information on molecular markers of drug sensitivity and drug response in cancer cells. It contains extensive drug sensitivity and genomic data sets that are important for the discovery of potential tumor therapeutic targets. The GDSC data came from 75,000 trials describing the reactions of about 200 anticancer drugs in more than 1,000 tumor cells. Our results demonstrated that 41 drugs exited significant differences in the high and low-risk groups.

In summary, we firstly performed WGCNA analysis in GSE63514 dataset and obtained 2306 module genes significantly associated with cervical cancer. Based on the TCGA-CESC dataset, 3892 differentially expressed genes (DEG1) were obtained, and 673 differentially expressed genes significantly correlated with cervical cancer were obtained, which were defined as “key genes”. And then based on the key genes, consensus clustering analysis, and differential expression analysis were performed in the TCGA-CESC dataset to obtain 804 differentially expressed genes (DEG2) in different cervical cancer subtypes. Twenty differentially expressed genes related to cervical cancer subtypes were obtained by cross-key genes and DEG2. After one-way and multivariate analysis of variance, we finally obtained three prognostic genes of APOD, APOC1 and SQLE significantly related to survival, and the expression of which was assayed in 10 cervical cancer tissue samples and 10 normal samples by RT-qPCR. A risk model involved in the three prognostic genes was then constructed along with validation according to the above-mentioned analysis in the present study. Based on the model, immune correlation and immunotherapy analyses were carried out, which will provide a theoretical basis and reference value for the exploration and treatment of cervical cancer. The limitations of our study were that the sample size for clinical validation of the three prognostic genes was small which would be improved in our further study.

## Conclusion

In the study, APOD, APOC1 and SQLE were identified as prognostic genes, and a prognostic genes-based risk model was constructed and validated, which will provide a novel viewpoint and reference value for the treatment and prognosis of cervical cancer.

## Supplementary Information

**Additional file 1**.** Figure S1**. The expression levels of the prognostic factors (APOD, APOC1 and SQLE) in TCGA-CESC dataset.

**Additional file 2**.** Figure S2**. The expression levels of the prognostic factors (APOD, APOC1 and SQLE) in GSE63514 dataset.

**Additional file 3**.** Figure S3**. Correlation of riskscore with prognostic factor. a Independent prognostic - univariate cox results of forest plots (Training set + Test Set). b Independent prognostic - multivariate cox results of forest plots (Training set + Test Set). c Independent prognostic - univariate cox results of forest plots (Validation set). d Independent prognostic - multivariate cox results of forest plots (Validation set).

**Additional file 4**.** Figure S4**. Nomogram was established with riskscore and T in TCGA-CESC dataset.

**Additional file 5**.** Figure S5**. Calibration curve of the nomogram.

**Additional file 6**.** Figure S6**. Decision curve analysis (DCA) curve of the nomogram.

**Additional file 7**.** Figure S7**. Correlation between immune cells and RiskScore.

**Additional file 8**.** Figure S8**. Median inhibitory concentration (IC50) of chemotherapy drugs in the high and low risk groups.

## Data Availability

The datasets generated and/or analysed during the current study are available in the Gene Expression Omnibus (GEO) repository, GSE63514 and GSE44001.
