# Supplementary material for: A risk prediction model mediated by genes of APOD/APOC1/SQLE associates with prognosis in cervical cancer
Source: BMC Womens Health. 2022 Dec 19;22:534. doi: 10.1186/s12905-022-02083-4 (PMC9764686; doi:10.1186/s12905-022-02083-4)

Figure S1 The expression levels of the prognostic factors (APOD, APOC1 and SQLE) in TCGA-CESC dataset.

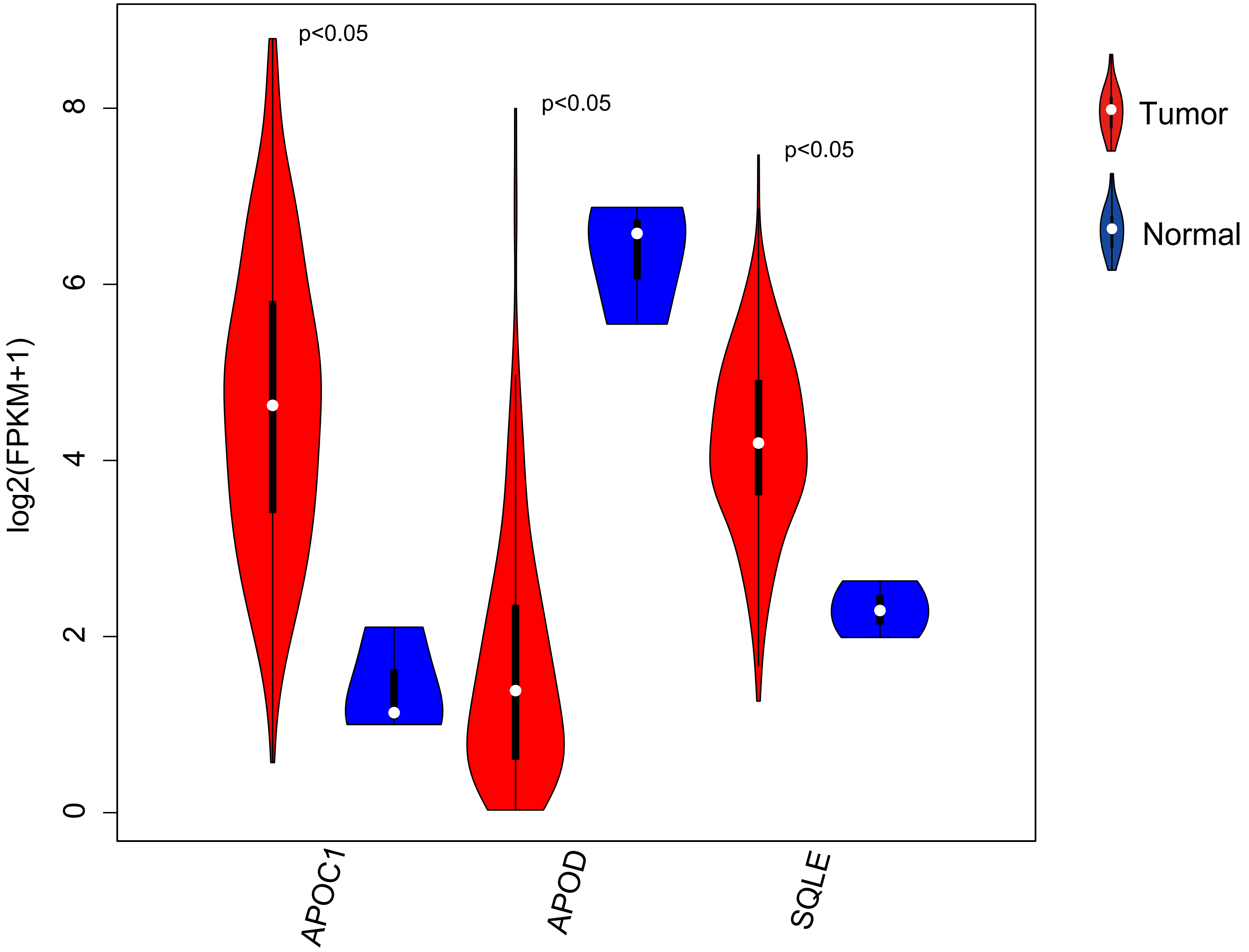

Supplement: Supplementary file 1 — Additional file 1. Figure S1. The expression levels of the prognostic factors (APOD, APOC1 and SQLE) in TCGA-CESC dataset. [file 12905_2022_2083_MOESM1_ESM.pdf]
