# Supplementary figures and images for: A risk prediction model mediated by genes of APOD/APOC1/SQLE associates with prognosis in cervical cancer
Source: BMC Womens Health. 2022 Dec 19;22:534. doi: 10.1186/s12905-022-02083-4 (PMC9764686; doi:10.1186/s12905-022-02083-4)

Figure S2 The expression levels of the prognostic factors (APOD, APOC1 and SQLE) in GSE63514 dataset.

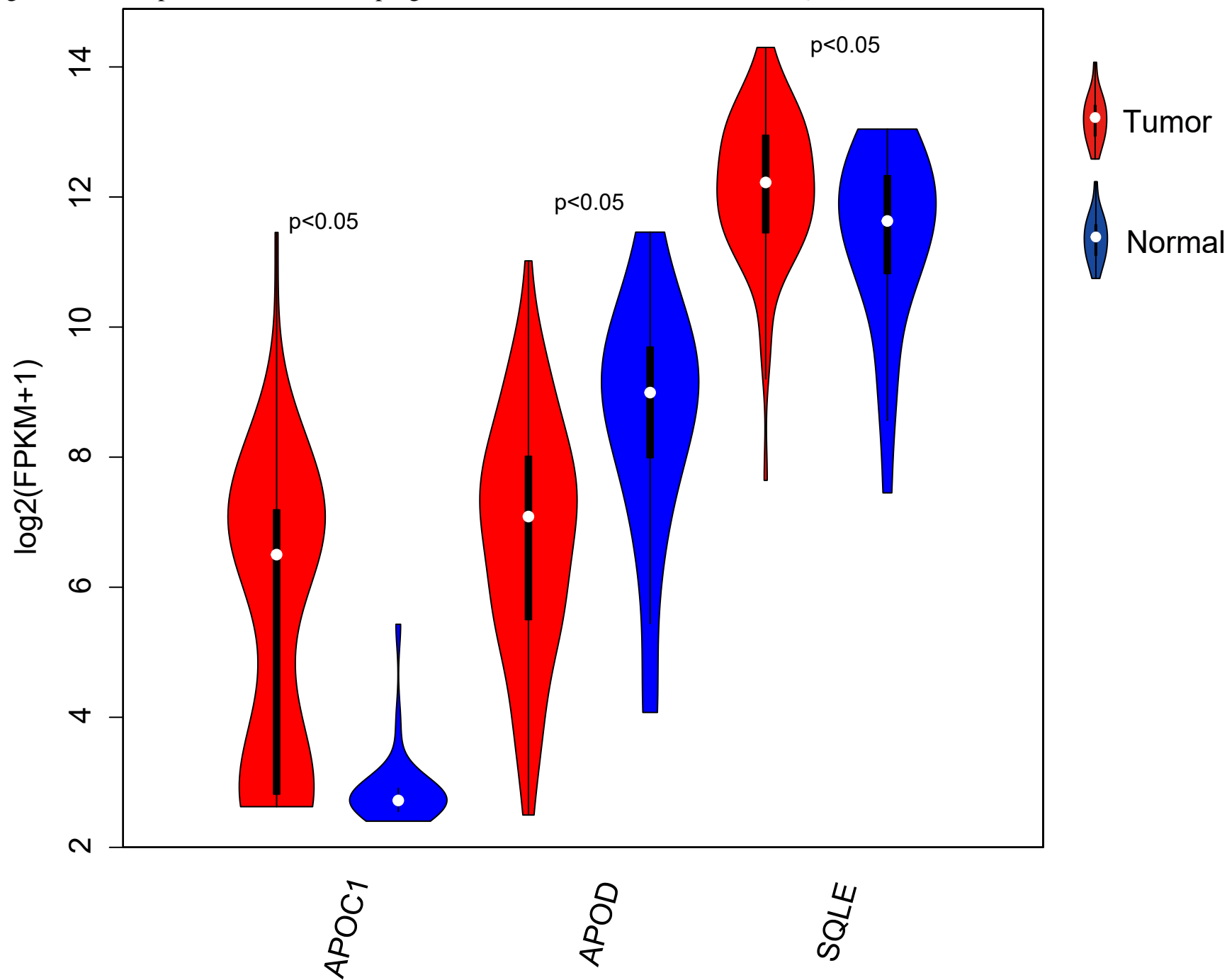

Supplement: Supplementary file 2 — Additional file 2. Figure S2. The expression levels of the prognostic factors (APOD, APOC1 and SQLE) in GSE63514 dataset. [file 12905_2022_2083_MOESM2_ESM.pdf]

Figure S4 Nomogram was established with riskscore and T in TCGA-CESC dataset.

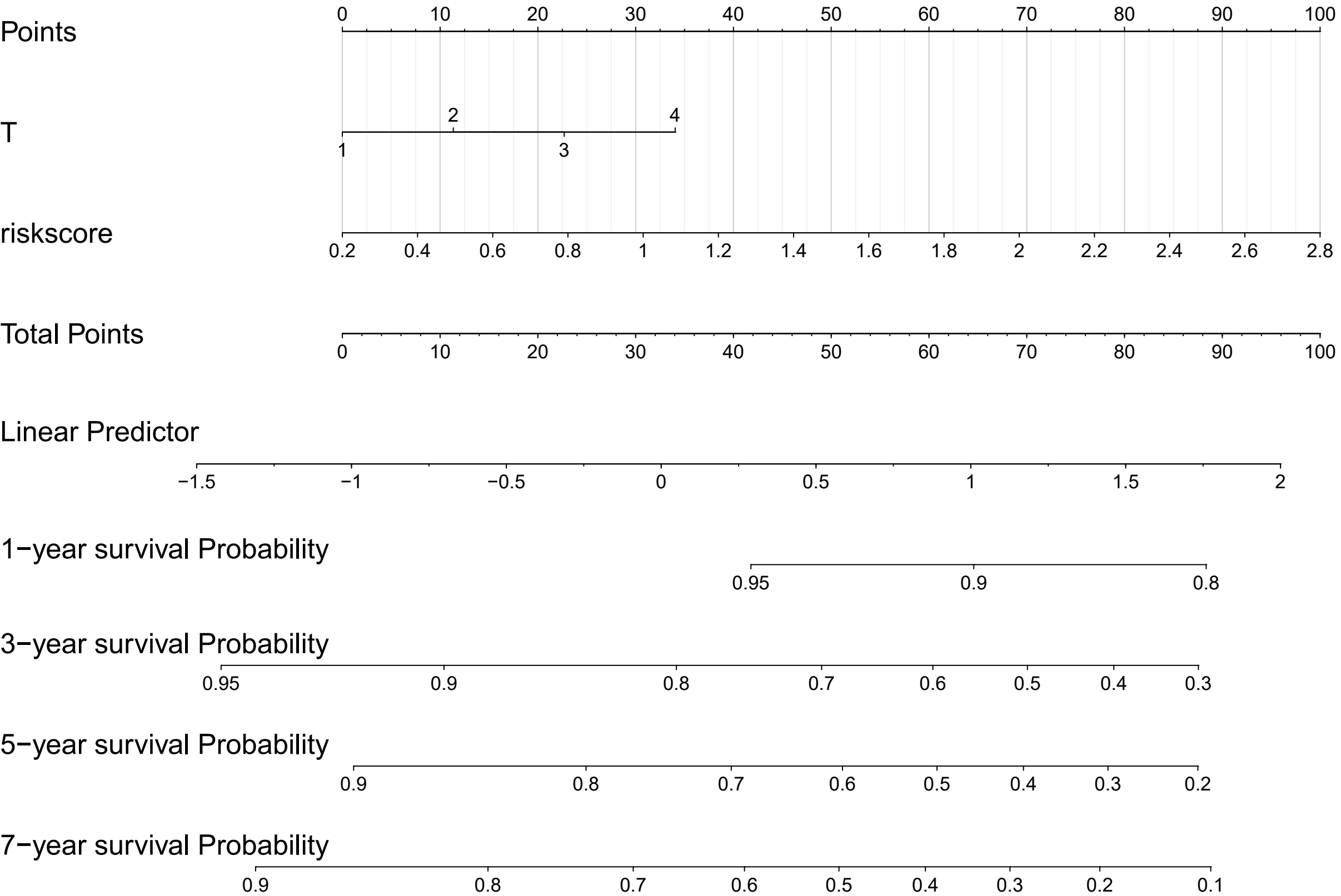

Supplement: Supplementary file 4 — Additional file 4. Figure S4. Nomogram was established with riskscore and T in TCGA-CESC dataset. [file 12905_2022_2083_MOESM4_ESM.pdf]

Figure S5 Calibration curve of the nomogram.

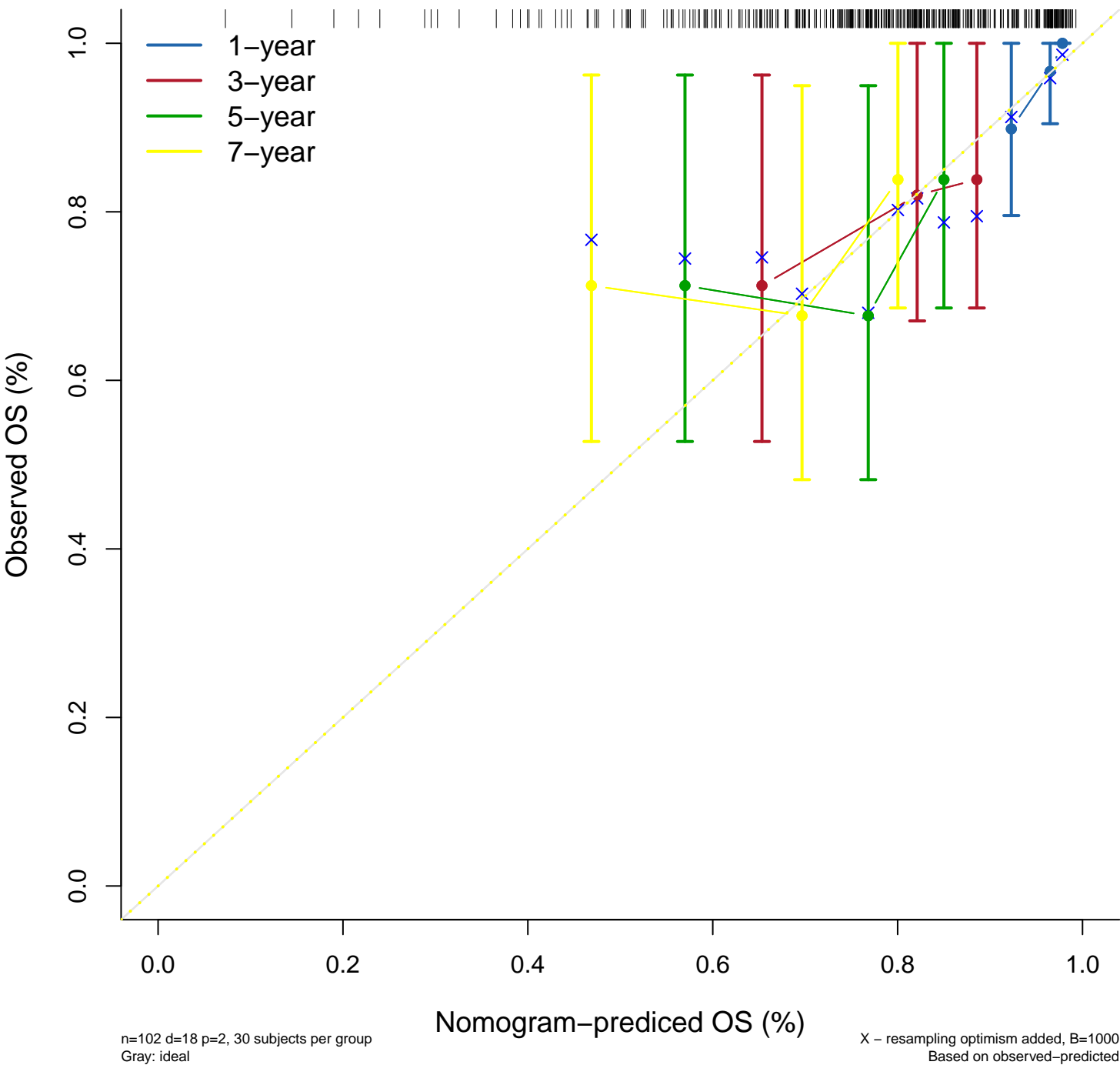

Supplement: Supplementary file 5 — Additional file 5. Figure S5. Calibration curve of the nomogram. [file 12905_2022_2083_MOESM5_ESM.pdf]

Figure S6 Decision curve analysis (DCA) curve of the nomogram.

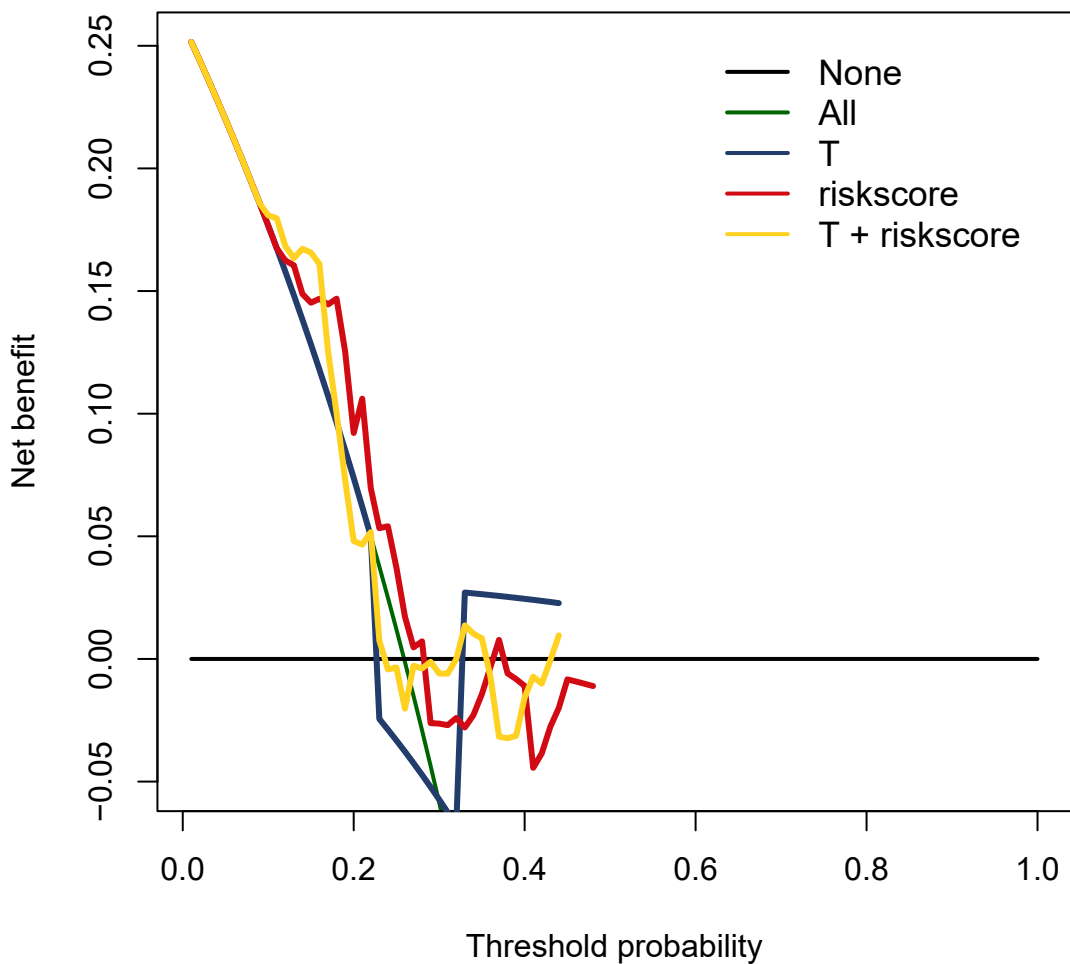

Supplement: Supplementary file 6 — Additional file 6. Figure S6. Decision curve analysis (DCA) curve of the nomogram. [file 12905_2022_2083_MOESM6_ESM.pdf]

Figure S7 Correlation between immune cells and RiskScore.

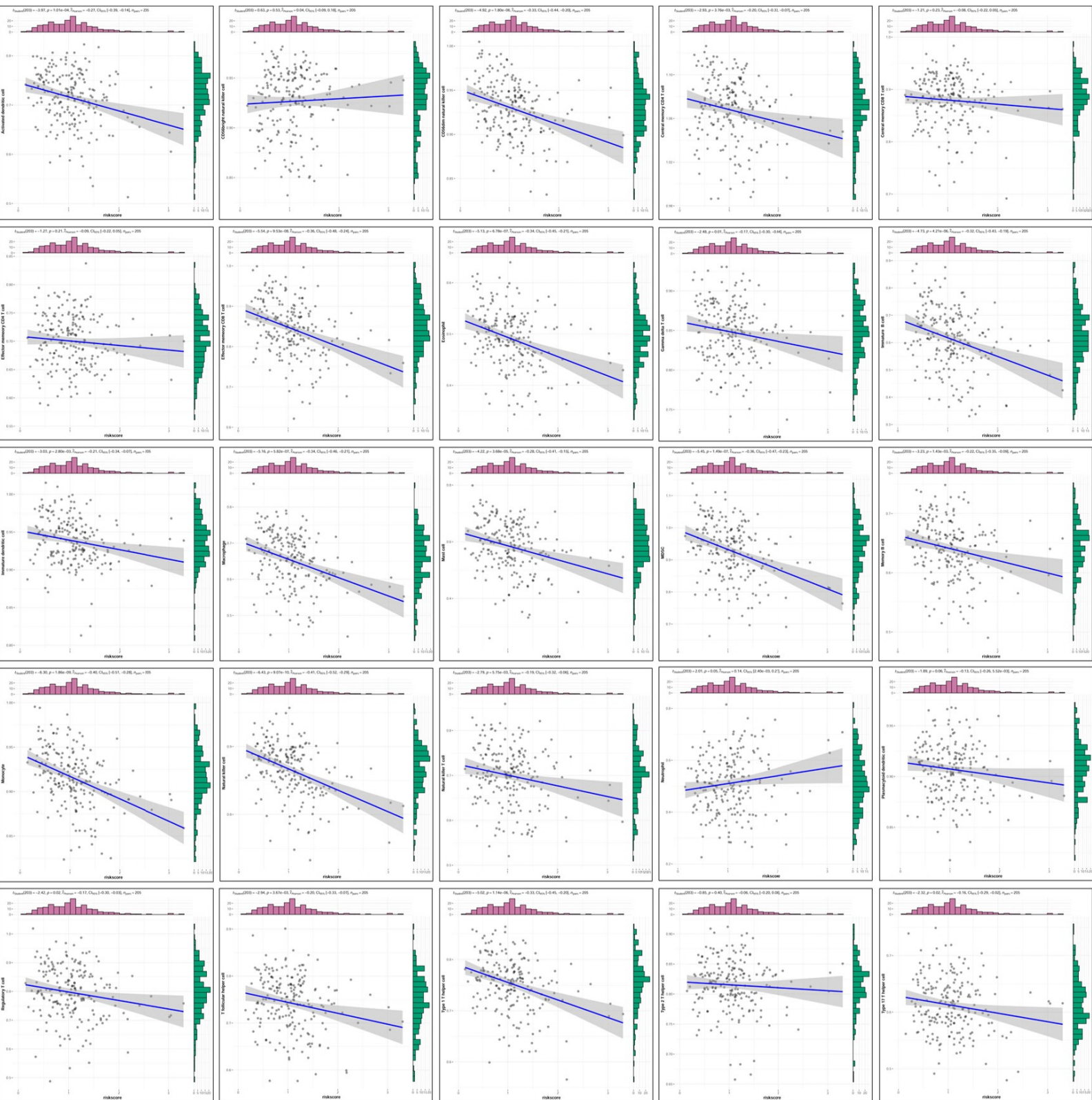

Supplement: Supplementary file 7 — Additional file 7. Figure S7. Correlation between immune cells and RiskScore. [file 12905_2022_2083_MOESM7_ESM.pdf]
