# Supplementary material for: A risk prediction model mediated by genes of APOD/APOC1/SQLE associates with prognosis in cervical cancer
Source: BMC Womens Health. 2022 Dec 19;22:534. doi: 10.1186/s12905-022-02083-4 (PMC9764686; doi:10.1186/s12905-022-02083-4)

Figure S3 Correlation of riskscore with prognostic factor. a Independent prognostic - univariate cox results of forest plots (Training set + Test Set).
b Independent prognostic - multivariate cox results of forest plots (Training set + Test Set).
c Independent prognostic - univariate cox results of forest plots (Validation set).
d Independent prognostic - multivariate cox results of forest plots (Validation set).

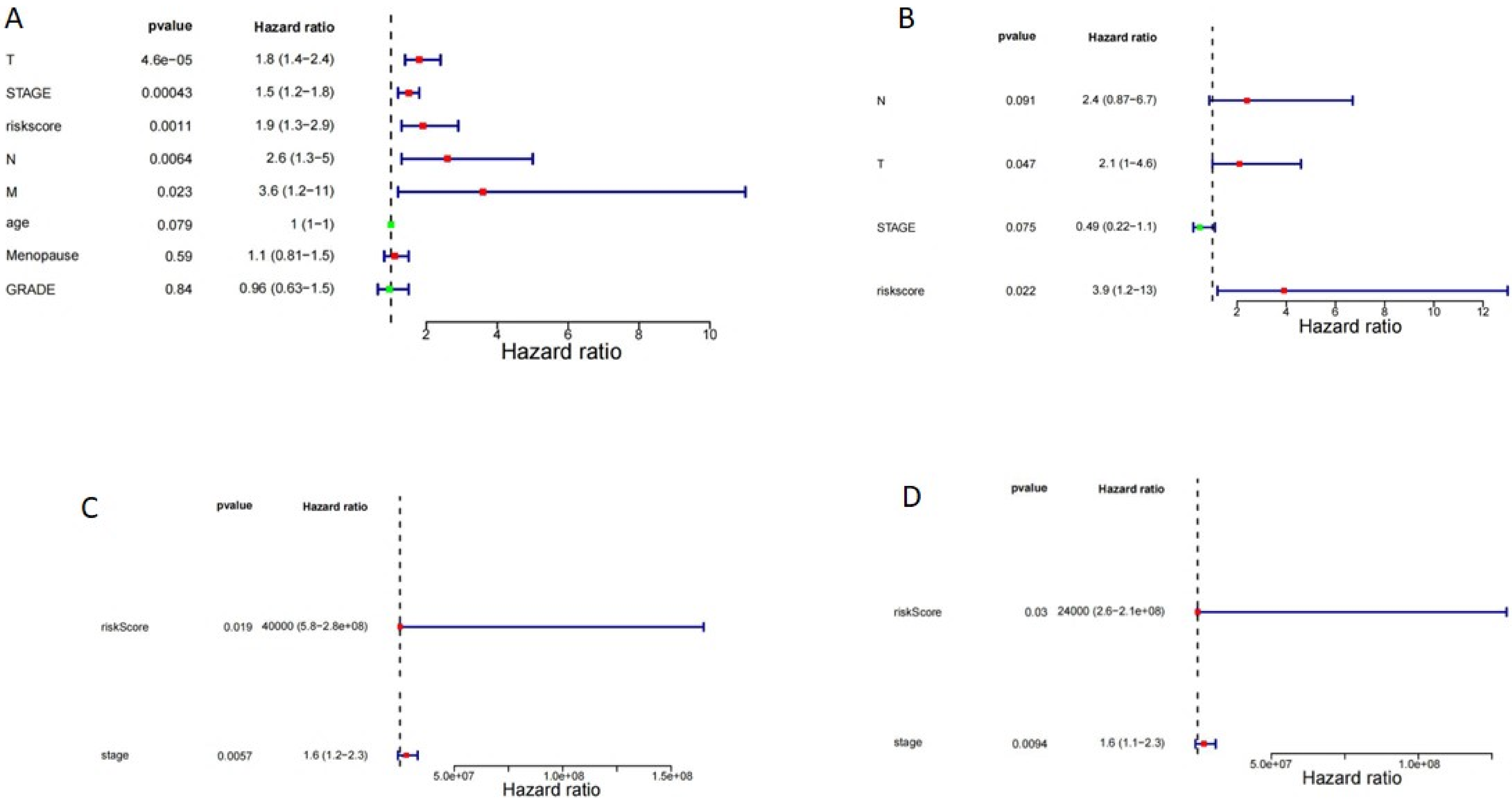

Supplement: Supplementary file 3 — Additional file 3. Figure S3. Correlation of riskscore with prognostic factor. a Independent prognostic - univariate cox results of forest plots (Training set + Test Set). b Independent prognostic - multivariate cox results of forest plots (Training set + Test Set). c Independent prognostic - univariate cox results of forest plots (Validation set). d Independent prognostic - multivariate cox results of forest plots (Validation set). [file 12905_2022_2083_MOESM3_ESM.pdf]
