# Supplementary material for: A risk prediction model mediated by genes of APOD/APOC1/SQLE associates with prognosis in cervical cancer
Source: BMC Womens Health. 2022 Dec 19;22:534. doi: 10.1186/s12905-022-02083-4 (PMC9764686; doi:10.1186/s12905-022-02083-4)

Figure S8 Median inhibitory concentration (IC50) of chemotherapy drugs in the high and low risk groups.

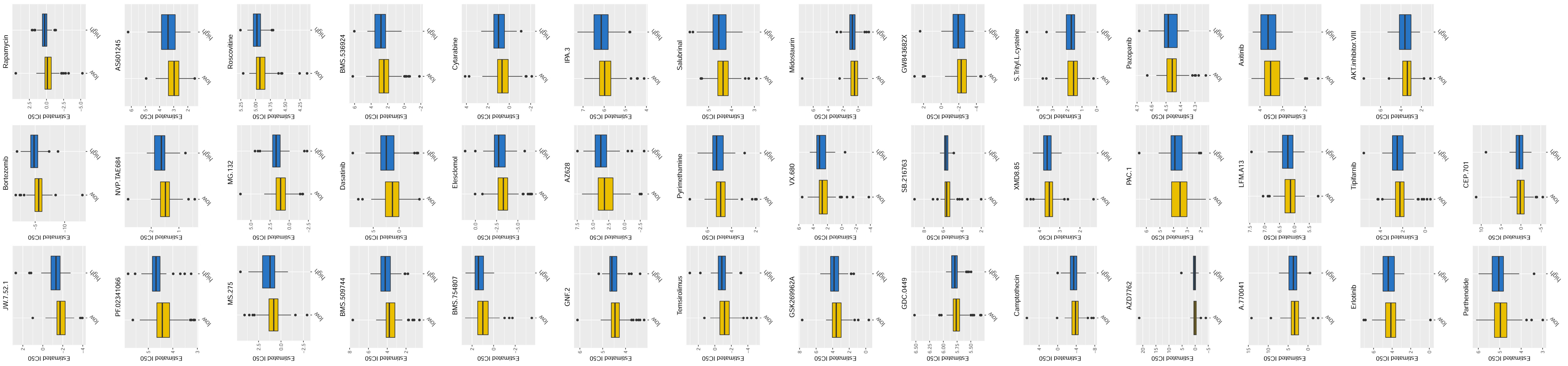

Supplement: Supplementary file 8 — Additional file 8. Figure S8. Median inhibitory concentration (IC50) of chemotherapy drugs in the high and low risk groups. [file 12905_2022_2083_MOESM8_ESM.pdf]
